# Cortical theta–gamma coupling governs the adaptive control of motor commands

**DOI:** 10.1093/braincomms/fcac249

**Published:** 2022-10-06

**Authors:** Rachel K Spooner, Tony W Wilson

**Affiliations:** Institute for Human Neuroscience, Boys Town National Research Hospital, Boys Town, NE, USA; College of Medicine, University of Nebraska Medical Center, Omaha, NE, USA; Institute of Clinical Neuroscience and Medical Psychology, Heinrich-Heine University, Düsseldorf, Germany; Institute for Human Neuroscience, Boys Town National Research Hospital, Boys Town, NE, USA; Department of Pharmacology and Neuroscience, Creighton University, Omaha, NE, USA

**Keywords:** cross-frequency, magnetoencephalography, motor execution, proactive, reactive

## Abstract

Motor control requires the adaptive updating of internal models to successfully target desired outcomes. This adaptive control can be proactive, such that imminent actions and corresponding sensorimotor programmes are anticipated prior to movement, or reactive, such that online error correction is necessary to adjust to sudden changes. While substantial evidence implicates a distributed cortical network serving adaptive control when behavioural changes are required (e.g. response inhibition), the neural dynamics serving such control when the target motor commands are to remain intact are poorly understood. To address this, we developed a novel proactive–reactive cued finger tapping paradigm that was performed during magnetoencephalography by 25 healthy adults. Importantly, to ensure condition-wise differences in adaptive cueing were not attributable to changes in movement kinematics, motor selection and planning processes were held constant despite changes in task demands. All data were imaged in the time-frequency domain using a beamformer to evaluate the effect of proactive and reactive cues on movement-related oscillations and subsequent performance. Our results indicated spectrally specific increases in low (i.e. theta) and high (i.e. gamma) frequency oscillations during motor execution as a function of adaptive cueing. Additionally, we observed robust cross-frequency coupling of theta and gamma oscillatory power in the contralateral motor cortex and further, the strength of this theta–gamma coupling during motor execution was differentially predictive of behavioural improvements and decrements during reactive and proactive trials, respectively. These data indicate that functional oscillatory coupling may govern the adaptive control of movement in the healthy brain and importantly, may serve as effective proxies for characterizing declines in motor function in clinical populations in the future.

## Teaser

Motor control is served by multispectral oscillatory responses in the primary motor cortex, but whether these responses are modulated by proactive versus reactive movement environments remains unknown. Spooner and Wilson report that cross-frequency coupling of theta and gamma oscillations are differentially predictive of behavioural performance during proactive and reactive movements.

## Graphical Abstract

**Graphical abstract:**
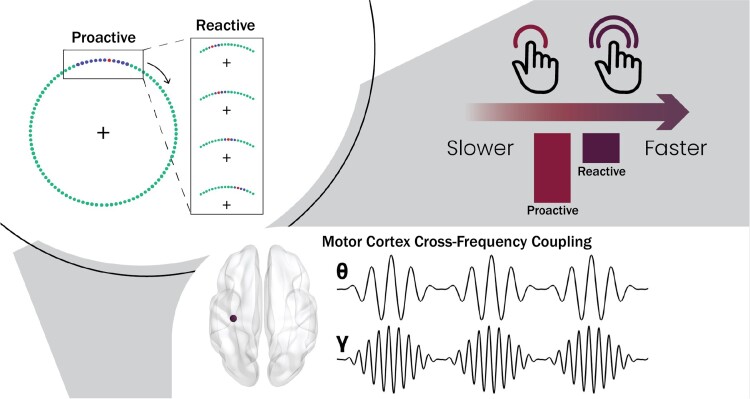

## Introduction

A basic tenant of volitional motor control is that desired movement kinematics are transformed into discrete plans to successfully execute and terminate motor actions. After repeated exposure to a particular action or movement sequence, an internal model is generated within the sensorimotor system, and this is continuously and adaptively updated to fulfil task demands.^1-4^ Such motor control can be proactive, such that internal models of intended actions are anticipated prior to movement cues, or it can be reactive, whereby these models are adjusted on the fly to account for ongoing changes in the goal parameters. Previous studies have primarily interrogated the proactive and reactive control of movement through the use of stop-signal or Go/No-Go task paradigms (i.e. response inhibition), whereby participants are instructed to respond to an expected cue (i.e. proactive) and to inhibit their response to an unexpected cue (i.e. reactive).^5,6^ Generally, these studies have used a variety of approaches (e.g. functional magnetic resonance imaging, lesion studies, local field potential recordings) to demonstrate better behavioural outcomes (e.g. shorter stop-signal reaction times, higher stopping accuracy), indicative of better *reactive* inhibitory control that is concomitant with the recruitment of an extensive cortical and subcortical network (e.g. greater relative power, increased network connectivity, shorter connectivity pathways) including hubs in the primary motor (M1) cortex, supplementary motor areas, premotor cortex, inferior frontal gyrus and basal ganglia during reactive trials compared with proactively cued trials.^7-12^ While these studies have been incredibly informative regarding the proactive and reactive mechanisms serving *motor inhibition*, the need to simply *adjust* movement parameters in real time is much more ecologically common than the need to suppress movement. However, the neural mechanisms serving such adaptive control when the motor command needs merely adjusted to achieve the target action are less well understood.

Volitional movements require a coordinated ensemble of spatiotemporally precise neural oscillations during the planning, execution and termination phases of movement. Specifically, decreases in oscillatory alpha and beta power are evident prior to and during movement execution, and are thought to reflect the active engagement of the motor network including hubs in bilateral M1, supplementary motor and parietal cortices.^13-16^ In contrast, increases in the beta range following movement offset are prevalent slightly anterior to the contralateral M1, and may simply reflect the return of beta oscillatory power to idling levels.^17,18^ Alternatively, this post-movement increase in power may reflect the functional inhibition of the motor system during motor termination, and/or sensory reafference to the motor cortex.^17,19-21^ Other spectral changes necessary for the execution of motor actions include lower (i.e. theta) and higher frequency oscillations (i.e. gamma). While the precise role of movement-related theta and gamma oscillatory responses are not well understood, transient increases in these responses may serve as temporal coordinators and robust execution signals for motor action in the contralateral M1 for theta^22,23^ and gamma,^20,24-27^ respectively.

Interestingly, while peri-movement oscillations in the alpha and beta range have been shown to be especially pertinent to higher order planning and movement selection factors, including response certainty^13,14,16,28,29^ and complexity,^30^ these oscillatory responses are less affected by the actual movement parameters themselves (e.g. force applied, frequency of movements).^31^ In contrast, high-frequency gamma activity seems to be particularly susceptible to changes in the kinematics, concomitant with alterations related to cognitive load when the movement to be performed is identical. For example, recent evidence suggests that the spectral properties of movement-related gamma activity (e.g. power and/or frequency) are robustly modulated by increased cognitive demand, including increased response interference and the reorienting of attention.^32-36^ Thus, oscillatory dynamics in the gamma range may provide valuable information regarding proactive and reactive control when the motor plan and selection processes are held constant. Herein, we developed a novel proactive and reactive finger tapping paradigm that was performed during magnetoencephalographic (MEG) imaging to evaluate the dynamic neural mechanisms serving the adaptive control of voluntary movement. Specifically, participants completed a single flexion-extension of the right index finger each time a red dot reached a target interval denoted in blue that surrounded the 12 o’clock position of a clock-like array (*proactive* condition). In the *reactive* condition, participants were presented with the same stimulus features as in the proactive condition, although when the red dot was a fixed distance away from the target interval (∼150 ms), the interval would shrink and shift to one of four locations within the original target interval’s overall span. Unlike previous work where movement parameters changed as a function of reactive cueing (e.g. inhibiting a pre-planned motor response, engaging different muscle groups, etc.), participants were instructed to respond identically, regardless of task condition. This allowed for more straightforward interpretations of adaptive cueing mechanisms (e.g. proactive versus reactive cues), as the to be performed movement was identical across both conditions. Importantly, our results reveal a dynamic coupling of cortical oscillations (i.e. theta and gamma) during motor execution in the contralateral M1 and further, robustly link the strength of cross-frequency coupling in M1 to the behavioural outcomes serving cue-related adaptations to control voluntary movements.

## Materials and methods

### Participants

Twenty-five healthy adults (12 females, *M*_age_ = 25.34 years, range 21–32 years old, all right handed) were recruited for this study. Exclusionary criteria closely followed that described in previous papers (e.g. diagnosed neurological or psychiatric disorder, medical illness affecting CNS function, history of head trauma or loss of consciousness, current substance use/dependence, ferromagnetic implants).^37,38^ Written informed consent was obtained following a full description of the study in accordance with the guidelines of the University of Nebraska Medical Center’s Institutional Review Board.

### MEG data acquisition and experimental paradigm

**Figure 1:**
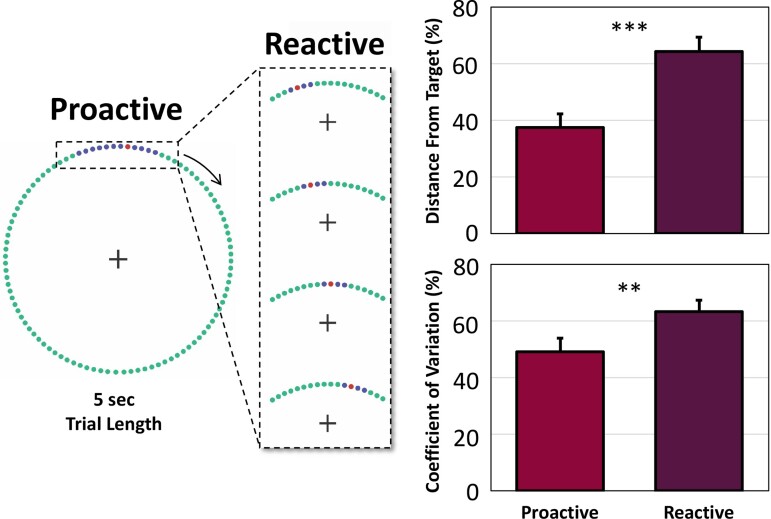
**Proactive–reactive motor control task and behaviour.** (Left): Participants fixated on a centrally located crosshair as a red dot moved clockwise towards the blue target interval. Participants were instructed to respond with their right index finger when the red dot was in the blue interval (proactive condition). In the reactive condition, participants were presented with the same proactive stimulus features (i.e. target interval parameters) and performed the same movement, but the blue target interval shrunk and shifted to one of four locations when the red dot reached a fixed distance away from the original target interval (∼150 ms). Trial types were presented in pseudorandomized order and the instructions were identical regardless of the target’s location. A video of the task paradigm is provided in the supplemental materials (Supplementary Video. 1). (Right): Behavioural analysis of response distance from target onset (normalized to the size of the target interval) revealed significantly longer distances (i.e. slower responses) during reactive trials compared with proactive ones (*t*(24) = 11.05, *P* < 0.001). In addition, participants were more variable in their response distances from target onset for reactive trials compared with proactive trials (*t*(24) = 3.04, *P* = 0.006). **P < 0.01, ***P < 0.001.

Our MEG data acquisition approach closely followed that described in previous papers using a MEGIN (MEGIN, Helsinki, Finland) 306-sensor instrument recorded at 1 kHz sampling rate (0.1–330 Hz acquisition bandwidth).^39-43^ Participants were seated in a nonmagnetic chair with their head positioned within the MEG helmet-shaped sensor array. To evaluate the proactive and reactive control of movement, we developed a novel finger tapping paradigm where participants were asked to perform the same flexion-extension of the right index finger to expected or unexpected stimulus targets. In the *proactive* condition, participants were instructed to maintain fixation on a centrally located crosshair while a red dot moved in a clock-like pattern towards a blue target interval that was fixed near the 12 o’clock position, and performed a single flexion-extension of the right index finger when the red dot was within the blue target interval (see *Proactive* in Fig. 1). This dot completed a full rotation around the clock-like circle (without tick marks or numbers) every 5 s, and was meant to serve as a pacing mechanism. For the *reactive* condition, participants were presented with the same proactive stimulus features of the clock-like design, however, when the red dot reached a fixed distance away from the original blue interval (∼150 ms), the target’s location (i.e. the blue interval) would shrink and shift within the original interval to one of four fixed locations (see *Reactive* in Fig. 1). This distance of ∼150 ms prior to the original target onset was carefully chosen to ensure participants did not anticipate the shift and new location of the target and thus, did in fact exhibit a reactive behavioural response. However, response instructions were identical regardless of the interval’s location (i.e. respond once when the red dot was within the blue target interval). Participants completed 135 *proactive* and 85 *reactive* trials and target interval locations were presented in a pseudorandom order. For a video demonstration of the experimental paradigm, see Supplementary Video. 1. Of note, this ratio of trials per experimental condition was based on previous literature evaluating proactive and reactive processing,^9,11,44^ which largely employs a 2:1 ratio of proactive to reactive trials, respectively to elicit the traditionally executed reactive behavioural response. The total time to complete the experiment was ∼18 min. Pairwise statistical tests of response distance from target onset and coefficient of variation were conducted to evaluate behaviour as a function of proactive or reactive motor control. All MEG data were individually corrected for head motion and subjected to noise reduction using temporal signal space separation.^45,46^

### Structural MRI processing and MEG coregistration

Prior to the MEG measurement, four coils were attached to the participant’s head and the locations of these coils, in addition to the three fiducial points and scalp surface, were determined with a 3D digitizer (Fastrak 3SF0002, Polhemus Navigator Sciences, Colchester, VT, USA). Once the participant was positioned for MEG recording, an electric current with a unique frequency label (e.g. 322 Hz) was fed to each of the coils to induce a measurable magnetic field that would allow each coil to be localized with respect to the sensor array throughout the recording session. All MEG measurements were then transformed into a common coordinate system and each participant’s MEG data were coregistered with T_1_-weighted structural magnetic resonance images prior to source space analyses using brain electrical source analysis (BESA) MRI (Version 2.0; BESA GmbH, Gräfelfing, Germany). Structural MRI data were aligned parallel to the anterior and posterior commissures and transformed into standardized space, along with the functional data, after beamforming (see below). Further details of our structural MRI processing and MEG coregistration pipeline can be found in recent papers.^37,38^

### MEG preprocessing, time-frequency transformation and sensor-level statistics

Cardiac and ocular artefacts (e.g. blinks, eye movements) were removed from the data using signal-space projection and the projection operator was accounted for during source reconstruction.^47^ Epochs were of 4500 ms duration, with 0 ms defined as movement onset and the baseline being the −2000 to −1500 ms window. Epochs containing artefacts were rejected based on a fixed threshold method, supplemented with visual inspection. On average, 72 trials per participant and condition remained after balancing trial numbers across task conditions (i.e. pseudorandom omission of ∼50 proactive trials throughout the entire experimental run) and subsequent artefact rejection.

Statistical analyses at the sensor level closely followed that described in previous papers.^39-43,48^ Specifically, following artefact rejection, epochs were transformed into the time-frequency domain using complex demodulation,^49^ and the resulting spectral power estimations per sensor were averaged over trials to generate time-frequency plots of mean spectral density. These sensor-level data were normalized to the baseline period (i.e. −2000 to −1500 ms time period). The specific time-frequency windows used for source reconstruction were determined by statistical analysis of the sensor-level spectrograms involving non-parametric permutation testing (i.e. 10 000 permutations) across all participants’ trials, task conditions and gradiometers.^50,51^

### MEG source imaging

Source reconstruction of cortical oscillatory networks were computed using the dynamic imaging of coherent sources (DICSs) beamformer,^52^ which uses the cross-spectral density matrices to calculate noise-normalized source power for the entire brain volume (i.e. per voxel) using baseline periods of equal duration and bandwidth.^53^ Normalized source power was computed over the entire brain volume per participant at 4.0 × 4.0 × 4.0 mm resolution for the time-frequency periods identified through statistical analysis of the sensor-level data. The resulting 3D brain images were averaged across all participants and task conditions to assess the neuroanatomical basis of the significant oscillatory responses identified through the sensor-level analysis, and to allow identification of the peak voxels per oscillatory response. Voxel time series data were then extracted from each participant’s data per condition using previously published methods.^37,38^ Once the voxel time series data were extracted, we computed the envelope of the spectral power within the frequency range used in the beamforming analysis. From this time series, we computed the relative (i.e. baseline-corrected) response time series of each participant per task condition at the single-trial level (see *Cross-frequency coupling* section below). MEG preprocessing and imaging used the BESA (Version 7.0) software. Further details of our source analysis pipeline can be found in our recent publications.^38-40,48^

To examine the effect of proactive and reactive stimulus cueing on movement-related neural dynamics, we conducted paired sample *t*-tests between experimental conditions for each oscillatory response computed using DICS reconstruction methods (i.e. pseudo *t*-values). All pairwise testing was conducted using *JASP*. Additionally, to complement traditional frequentist hypothesis testing approaches, Bayesian analyses using zero-centred Cauchy distributions were also conducted in *JASP* to evaluate evidence for the data favouring the null/alternative hypotheses.

### Cross-frequency coupling and statistical analyses

We also interrogated the functional coupling of relevant oscillatory responses modulated by adaptive cueing in the motor network (i.e. theta and gamma oscillatory power). First, we extracted relative single-trial voxel time series data (i.e. baseline corrected) from the peak voxels per oscillatory response during movement onset (i.e. −125 to 125 ms for theta; −100 to 100 ms for gamma) to conduct condition-wise multilevel models (MLMs) in R following standard data evaluation and trimming procedures (i.e. excluded cases exceeding 2.5 standard deviations from the participant’s mean). Specifically, we defined relative theta oscillatory power (continuous), task condition (factor with two levels) and their interaction as fixed effects, with subject and trial number defined as nested random effects to predict relative gamma oscillatory power at the single-trial level. Next, we aimed to directly link the strength of theta–gamma cross-frequency coupling in the motor cortex to behavioural performance on the task. Specifically, we computed the predicted gamma oscillatory power using the regression equation modelling significant theta–gamma power-to-power coupling at the single-trial level (see above). The resulting predicted relative gamma power reflects M1 gamma oscillatory power *accounting for* observed levels of theta power. Next, we ran an MLM with predicted gamma power (continuous), task condition (factor with two levels) and their interaction as fixed effects, with subject and trial number as nested random effects to predict the response distance from target onset (ratio score). Importantly, MLMs were conducted using the *lme4* package in R and corrected for multiple comparisons using Tukey’s multiple comparison test.

Finally, recent evidence suggests that the nesting of higher frequency oscillations (i.e. gamma) within lower frequency signatures (i.e. theta) may not only be functionally coordinated by neural amplitudes, but also, by dynamic phase–amplitude interactions.^54^ Thus, for completeness, we evaluated whether theta–gamma *phase*–*amplitude* coupling (PAC) was present in the current dataset and further, whether it was modulated by reactionary motor control. Thus, we implemented the event-related phase–amplitude coupling method proposed by Voytek and colleagues,^55^ which expands upon traditional estimations of PAC in electrophysiological data to extract the correlation between higher frequency amplitudes and lower frequency phases per time point, thus not obscuring the temporal evolution of task-dependent PAC. We calculated the correlation of gamma (i.e. 70–80 Hz) power with theta (i.e. 4–8 Hz) phase at each time point in our experiemental epoch (i.e. −2000 to 2500 ms) for each condition separately (i.e. proactive versus reactive) using voxel time series extracted from the contralateral M1. The resulting PAC values range from 0 to 1, with larger values indicative of greater theta–gamma PAC in M1. Next, we conducted pairwise *t*-tests of task condition and Bayesian statistics to evaluate whether M1 theta–gamma PAC differed by adaptive cueing during motor execution (i.e. −125 to 125 ms window) using *JASP*.

### Data availability

Data from this study will be made available to qualified researchers upon reasonable request to the corresponding author.

## Results

### Behavioural performance

All 25 participants were able to successfully complete the quasi-paced finger tapping paradigm during MEG (Fig. 1). To evaluate the proactive and reactive control of motor performance on the task, we assessed measures of reaction time (i.e. response distance from target interval onset) and the coefficient of variation to assess the variability in behavioural performance, given the quasi-paced nature of the task instructions (see *Experimental paradigm*). Specifically, we computed ratio scores for both behavioural metrics to account for the difference in interval size between proactive and reactive conditions (e.g. response time distance from target onset/length of total target interval). Thus, all behaviours are expressed as per cent distance and/or variability from the onset of the blue target interval. Interestingly, participants responded significantly slower (*t*(24) = 11.05, *P* < 0.001) and with greater response time variability (*t*(24) = 3.04, *P* = 0.006) relative to the onset of the blue target interval for reactive trials compared with proactive trials (Fig. 1).

### Sensor-level analysis

**Figure 2:**
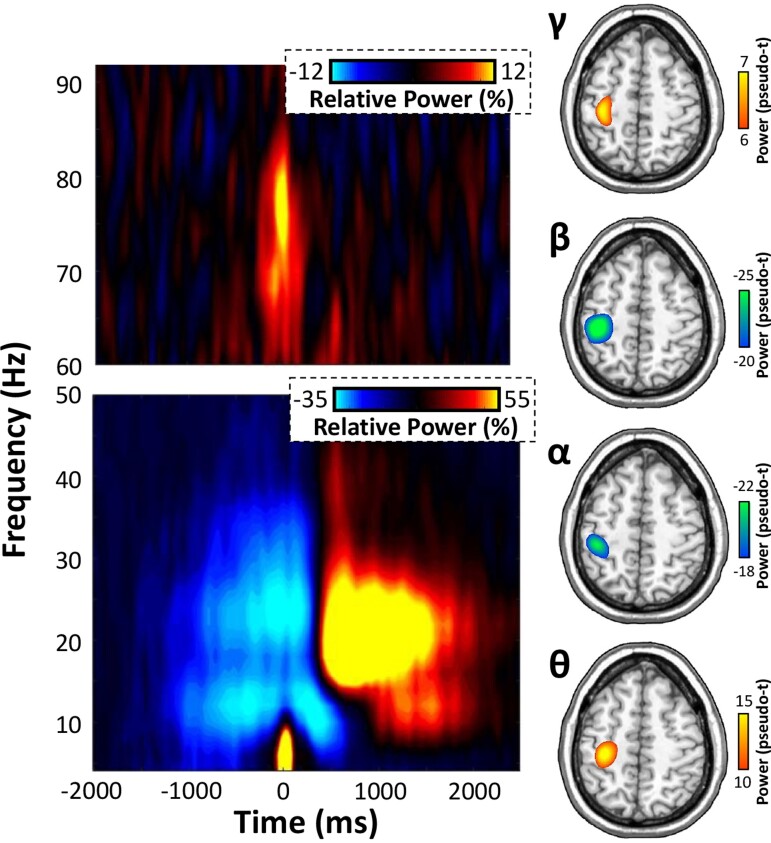
**Sensor- and anatomical-space movement-related oscillations.** (Left): Time-frequency spectrograms locked to the onset of the movement (0 ms) for two sensors near the left sensorimotor cortex. The *x*-axis denotes time (in ms) and the *y*-axis represents frequency (in Hz). Relative power (i.e. percent change from baseline −2000 to −1500 ms) is expressed according to the colour scale bars inset in the top right of each spectrogram. (Right): Significant movement-related oscillations were imaged using a beamformer. Strong decreases in alpha (10–14 Hz) and beta (20–28 Hz) activity occurred 250 ms prior to and following movement, and these localized reliably to the left M1. In contrast, robust increases in theta (4–8 Hz) and gamma (70–80 Hz) activity were observed in the left M1 during movement onset (−125 to 125 and −100 to 100 ms for theta and gamma, respectively).

Time-frequency analyses indicated significant peri- and post-movement oscillatory responses in the theta (4–8 Hz), alpha (10–14 Hz), beta (20–28 Hz) and gamma (70–80 Hz) ranges. These responses were robust in gradiometers near the contralateral sensorimotor strip across all participants and both experimental conditions (*P* < 0.001, Fig. 2). Specifically, transient increases in theta and gamma oscillatory activity coincided closely with movement onset (theta: −125 to 125 ms; gamma: −100 to 100 ms), while decreases in alpha and beta activities were more temporally sustained surrounding the movement (i.e. −250 to 250 ms for alpha and beta). Finally, there was a robust synchronization in the beta range (16–26 Hz) that was detected during the 400–900 ms window following motor execution.

### Oscillatory signatures of proactive and reactive movement

**Figure 3:**
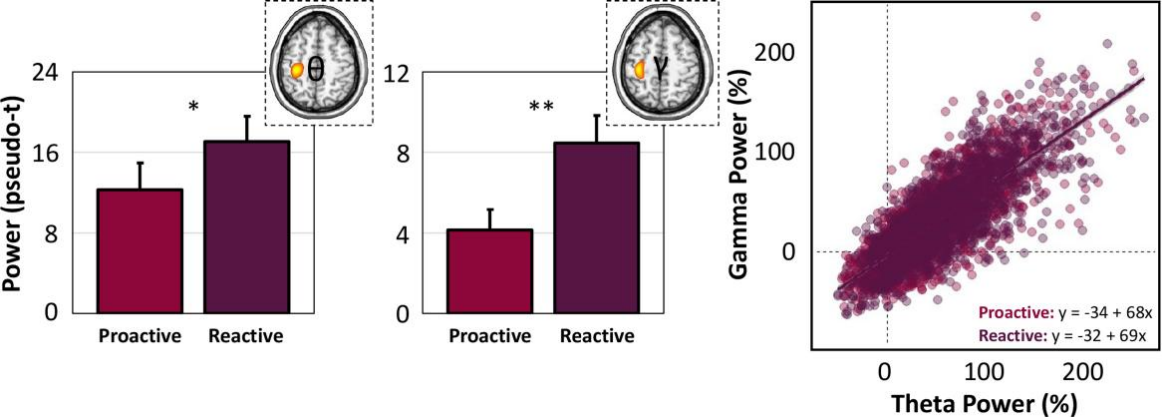
**Reactionary movement cues modulate theta and gamma oscillations.** (Left): Pairwise *t*-tests of task condition (i.e. proactive and reactive cues) on movement-related oscillations revealed robust increases in theta (4–8 Hz; *t*(24) = −2.27, *P* = 0.035) and gamma (70–80 Hz; *t*(24) = −2.87, *P* = 0.009) oscillatory power in the contralateral M1 during reactive trials compared with proactive ones. (Right): A single-trial linear mixed effects model of theta oscillatory power and task condition on gamma oscillatory power during movement onset was conducted to evaluate cross-frequency coupling in our data. There was a main effect of theta oscillatory power (i.e. baseline corrected) on relative gamma power (*F*(2173) = 6715.59, *P* < 0.001), such that increased theta power in the left M1 was significantly predictive of increased gamma power in the same region. There was no main effect of condition (*F*(3460) = 0.41, *P* = 0.501), nor a significant theta by condition interaction on gamma oscillatory power (*F*(3461) = 0.24, *P* = 0.627), suggesting that task condition did not modulate theta–gamma power-to-power coupling in the left M1. 95% confidence intervals are displayed for each regression line, although the slopes were nearly identical for each condition (see regression equations in bottom right of the scatterplot). Each point represents a single trial. *P < 0.05, **P < 0.01.

To identify the neural origins of oscillations seen at the sensor level, these windows were imaged using a beamformer. The resulting maps indicated that all five oscillatory responses reliably localized to the left M1 cortex contralateral to movement, regardless of experimental condition (Fig. 2). As described in the methods, we next extracted peak oscillatory activity from each respective grand-averaged cluster (i.e. across all participants and task conditions) in the M1 cortex for proactive and reactive trials separately to examine the effect of adaptive control of movement using pairwise comparisons and Bayesian statistics. Movement-related theta and gamma oscillatory activities were significantly stronger in response to reactive trials compared with proactive ones (Fig. 3), while event-related decreases in the alpha and beta range were unaffected by reactive cueing (theta: *t*(19) = −2.27, *P* = 0.035; alpha: *t*(23) = −0.59, *P* = 0.558; beta: *t*(23) = 1.01, *P* = 0.321; gamma: *t*(23) = −2.87, *P* = 0.009). Similarly, post-movement beta rebound (PMBR) responses following motor execution exhibited no differences as a function of task condition (*t*(24) = −0.34, *P* = 0.735). In fact, there was moderate evidence for the null hypothesis for each oscillatory response that was unaffected by the different task conditions, providing further evidence that reactive cueing does not modulate movement-related oscillations in the alpha and beta range (alpha: BF_01_ = 3.97, error % = 0.040; beta: BF_01_ = 2.94, error % = 0.0001; PMBR: BF_01_ = 4.49, error % = 0.030).

### Cross-frequency coupling differentially predicts proactive and reactive movements

Given the spectrally specific alterations in both low (i.e. theta) and high (i.e. gamma) frequency oscillations as a function of proactive and reactive movement cueing, we evaluated the dynamic coupling of these oscillations within the M1 cortex. Importantly, there is converging evidence from several lines of work implicating the functional coupling of theta and gamma oscillations to support local and network-level neuronal communication during cognitive and sensory processing.^22,54,56-62^ Thus, to examine the specific impact of theta–gamma coupling during proactively and reactively cued simple finger movements, we conducted an MLM in R using single-trial data. As described in the methods, we extracted the time series of the peak voxel in left M1 and computed the spectral envelope for theta and gamma oscillatory responses at the single-trial level. Theta oscillatory power (continuous), task condition (two levels) and their interaction were treated as fixed effects, with subject and trial number as a nested random effect to predict gamma oscillatory power in the motor cortex. Our results indicated a significant main effect of theta power on gamma power, such that increases in theta power were predictive of increases in gamma power, indicative of significant power-to-power cross-frequency coupling during movement execution in left M1 (*F*(2173) = 6715.59, *P* < 0.001; Fig. 3). In contrast, there were neither main effects of task condition nor a significant theta by condition interaction (*P* > 0.501) on gamma oscillatory power.

For completeness, we next evaluated whether adaptive cueing modulated dynamic coupling of theta and gamma oscillations via phase–amplitude interactions, as opposed to the power-to-power interactions described above. As described in the methods, we implemented the event-related phase–amplitude coupling approach^55^ and conducted pairwise testing as a function of task condition. Our results indicated no significant differences in theta–gamma phase–amplitude coupling in M1 during motor execution, with moderate evidence for the null hypothesis observed (*M*_proactive_ = 0.15, SD_proactive_ = 0.03; *M*_reactive_ = 0.14, SD_reactive_ = 0.02; *t*(24) = 1.20, *P* = 0.241, BF_01_ = 2.48, error % = 0.00007).

**Figure 4:**
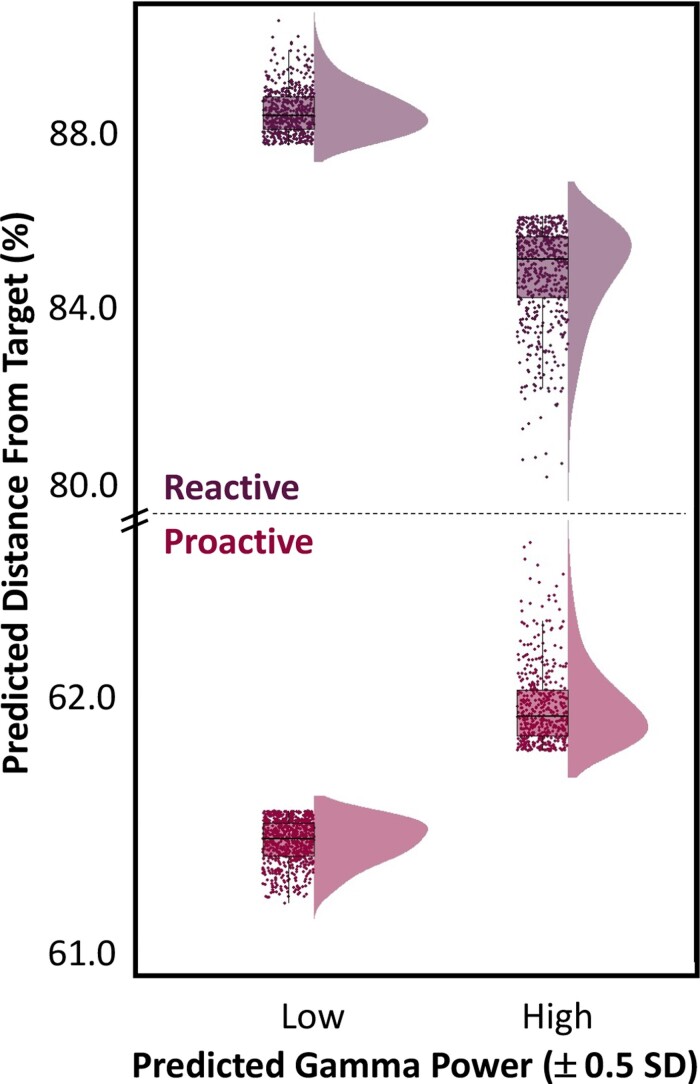
**Theta–gamma cross-frequency coupling modulates motor control.** A single-trial linear mixed effects model of theta–gamma coupling metrics, task condition and their interaction on response distances from target was conducted. Specifically, predicted gamma oscillatory power and task condition were treated as fixed effects, with subject and trial number treated as a nested random effect. There was a significant main effect of predicted gamma power (i.e. theta–gamma coupling metric; *F*(2263) = 6.86, *P* = 0.009), task condition (*F*(3015) = 512.61, *P* < 0.001) and a significant predicted gamma by condition interaction (*F*(3139) = 4.51, *P* = 0.034). Of note, predicted gamma power is the oscillatory power within the gamma range *accounting for* observed levels of theta oscillatory power and its regression coefficient (i.e. strength of the relationship between theta and gamma oscillations in M1). Likewise, predicted distance from target reflects response distances *accounting for* raw, observed levels of M1 theta–gamma oscillatory coupling. The raincloud plots show the predicted response distance from target (using the regression equation derived from the full model) on the *y*-axis and low and high levels of predicted gamma power (i.e. predicted levels of gamma by theta) on the *x*-axis. For the proactive condition, when theta–gamma power-to-power coupling was high in the contralateral M1, participants were slower to respond once the dot entered the target interval. In contrast, for reactive trials, increased theta–gamma coupling in the left M1 was predictive of faster response times relative to target onset.

Finally, we assessed whether the strength of theta–gamma power-to-power coupling in left M1 was predictive of behavioural performance on our motor task. To address this, we computed the predicted gamma power using the regression equation derived from our theta–gamma power-to-power coupling relationship (see model above). Briefly, because we did not observe any significant effect of condition, nor a theta by condition interaction in our MLM, we used the regression equation solely interrogating the predictive capacity of theta oscillatory power on gamma oscillatory power, as the regression coefficients did not differ from one another (see *Materials and methods*). Thus, we conducted a linear mixed effects model of predicted gamma power (i.e. indicative of theta–gamma power-to-power coupling; continuous variable), task condition (two levels) and their interaction as fixed effects, with subject and trial number as a nested random effect to predict response distance from target onset. Importantly, we observed a significant main effect of predicted gamma power (i.e. gamma activity predicted by theta activity in M1), such that increases in predicted gamma power during movement onset were predictive of closer response distances (i.e. faster responses; *F*(2263) = 6.86, *P* = 0.009). Additionally, as expected based on our behavioural analyses, there was a significant main effect of condition, such that response distances from target were significantly longer for reactive trials compared with proactive ones (*F*(3015) = 512.61, *P* < 0.001). Finally, there was a significant predicted gamma power by condition interaction, such that during proactive trials, higher levels of predicted gamma power in the motor cortex were associated with increased response distances, while for reactive trials, greater predicted gamma power (i.e. greater theta–gamma coupling) was predictive of faster response times relative to the onset of the target interval (*F*(3139) = 4.51, *P* = 0.034; Fig. 4). Notably, this interaction effect also held when gamma power was included as a covariate of no interest in the MLM.

## Discussion

Using a novel proactive–reactive movement paradigm and advanced oscillatory analyses, we interrogated the functional coupling of motor cortical oscillations serving the adaptive control of voluntary movements. Specifically, we observed robust decrements in task performance, such that during reactive trials (i.e. an unexpected change in target interval location), participants responded more slowly and were more variable compared with the expected, proactively cued targets. In regard to the brain, during movement onset, we observed significant increases in low- (i.e. theta) and high- (i.e. gamma) frequency oscillatory activity during performance of reactive trials compared with proactive ones, while peri- and post-movement oscillatory activity in the alpha and beta range were unaffected by conditional cues. Finally, our novel finger tapping task elicited robust cross-frequency power-to-power coupling in the left M1 contralateral to movement, such that during motor execution, theta–gamma coupling was strong and further, increases in theta–gamma cross-frequency coupling were predictive of better task performance during adaptive motor control. Below, we discuss the implications of these novel findings for understanding the proactive and reactive control of voluntary movement.

Previous investigations of proactive and reactive control of movement have employed a variety of experimental paradigms (e.g. stop-signal task, Go/No-Go) to examine movement selection and inhibition processes. These paradigms generally involve manipulating conditional cues unexpectedly (e.g. presented 25% of the time) and require participants to select a different movement (e.g. move a different finger) or alternatively, to inhibit the pre-planned movement. Importantly, these studies indicate that when movement cues are abruptly changed and participants must inhibit and/or significantly alter the parameters of the movement, behavioural performance is often degraded. For example, classic stop-signal paradigms tend to elicit key behavioural outcomes, including increased error rates to STOP trials, longer stop-signal response times (i.e. time needed to abort a pre-planned response) and even shorter response times to STOP trials when the action was completed erroneously.^9,11,44^ In contrast, for paradigms requiring motor responses to be altered based on a conditional cue (e.g. change in movement direction, different muscle groups engaged), response times tend to increase, indicative of poorer task performance.^13,63^ In contrast to these studies, the paradigm used in the current study enabled motor planning and selection processes to remain constant despite changing task demands, and thus facilitates a more direct interpretation of the findings. Interestingly, we found significant declines in behavioural performance during unexpected (i.e. reactive) cue trials, such that participants were slower to respond and much more variable in their responses (i.e. greater coefficient of variation) compared with the expected, proactively cued trials. Importantly, our results corroborate the previous work suggesting that reactive or unexpected cues directly affect motor outcomes compared with proactive ones. However, since prior work also involved changes to the movement (i.e. response inhibition, movement direction), the current findings are able to more directly link the alterations in performance to the reactive versus proactive dimension.

In regard to the brain, our novel proactive–reactive motor task elicited robust oscillatory activity in the theta, alpha, beta and gamma bands and further, we observed frequency-specific alterations in motor cortical oscillations as a function of task condition. Specifically, we observed stronger theta and gamma oscillations during motor execution in reactive compared with proactive trials, while peri-movement decreases in the alpha and beta range were unaffected by task condition. While the precise role of theta and gamma oscillations in motor control still requires careful consideration, emerging data indicates an important, temporally defined role in the execution of motor commands. For example, theta oscillations have long been seen as temporal coordinators and organizers of sensory information.^54,64-66^ This specific role appears to be ubiquitous across sensory cortices and may be further propagated to serve as coordinators of the precise timing of motor actions in the contralateral M1.^22^ In contrast, gamma oscillations in the motor cortex were primarily thought to reflect pro-kinetic signals that are particularly sensitive to basic movement parameters such as the force, frequency and pacing of the performed action.^25,67^ However, recent work by our laboratory and others have demonstrated alterations in movement-related gamma power and/or frequency with increased cognitive interference and attentional reallocation,^32-36^ suggesting that gamma oscillations in the contralateral M1 are susceptible to higher order control mechanisms, especially when the specific movement remains constant across changing cognitive demands. Critically, this idea of cognitively controlled, movement-related gamma synchrony is strengthened by the current data, as our task was designed to control for precise movement kinematics in order to fully disentangle the cueing mechanisms serving adaptive motor control.

Our most important finding was likely the dynamic, single-trial coupling of theta and gamma oscillations during motor execution in the contralateral M1 cortex. Specifically, single-trial analyses indicated that increases in theta oscillatory power robustly predicted increases in gamma oscillatory power in the same region and further, gamma power differentially predicted trial-by-trial behavioural outcomes as a function of task condition. Such cross-frequency coupling of neural oscillations may serve an important mechanistic role in coordinating the local- and network-level communication among neuronal pools oscillating at disparate rates. In particular, such functional coupling tends to give rise to a nesting of faster oscillations (e.g. gamma) that are temporally coordinated with and modulated by the phase and/or amplitude of slower oscillations (e.g. theta) to further govern behavioural performance, albeit the dynamic coupling observed in the current study was specific to power-to-power dynamics rather than phase–power interactions between lower and higher frequency oscillators.^54,58^ For example, the coupling of theta and gamma oscillations both locally and across the cortex has been shown to be critical for a variety of cognitive processes including memory,^57,59^ cognitive interference,^62^ associative learning,^56^ and motor function.^22,60,68^ In regard to the motor system, studies have shown substantial theta–gamma coupling in the rat motor cortex during rest and while moving (e.g. treadmill running,^60^ lever pulling^22^) and further, this phenomenon appears to be temporally synced with movement initiation. Importantly, our study is the first to evaluate theta–gamma cross-frequency coupling within the motor cortex during adaptive motor control. Additionally, while holding the actual movement constant, we demonstrated a condition-specific modulation of behavioural performance, such that gamma oscillatory power estimated by the levels of theta power during motor execution significantly predicted key parameters of the behavioural response. Specifically, during proactive trials where the target interval was expected, increases in predicted gamma power were significantly predictive of slower response times. In contrast, during reactive trials where the target interval change was unexpected, increased predicted gamma power was predictive of faster response times, which suggests that greater theta–gamma cross-frequency coupling in the motor cortex leads to enhanced adaptive motor control when real time parameter adjustments are needed. While behavioural decrements in response to increased M1 theta–gamma coupling during proactive trials seems counterintuitive at first glance, such findings would be predicted based on prior work in healthy young and older adults. For example, a recent study in our laboratory used an Eriksen Flanker paradigm during MEG to evaluate the role of motor cortical oscillations on the well-established age-related decline in visual selective attention function. The results demonstrated that M1 gamma power was a unique predictor of behavioural performance and further, in younger adults, elevations in M1 gamma power predicted faster reaction times regardless of task difficulty. In contrast, older adults did not exhibit this same behavioural benefit, and elevations in M1 gamma power were related to decrements in motor performance both in the presence and absence of visual interference, likely reflecting exhausted neural resources with increasing age.^39^ Such findings of elevated M1 gamma power contributing to behavioural benefits in times of increasing task difficulty compared with less difficult tasks are also corroborated by numerous studies in healthy young adults regarding response interference (e.g. multisource interference: Simon and Flanker stimuli)^36^ and attentional reorienting.^35^

## Conclusion

The current study was the first to identify the spectrotemporal dynamics serving adaptive motor control in the M1 cortex in healthy adults. Specifically, we developed a novel proactive–reactive movement paradigm and observed spectrally specific alterations in motor cortical oscillations during proactive and reactive movements and further, observed a dynamic coupling of theta and gamma oscillations during motor execution. Our study was also the first to directly link the strength of theta–gamma cross-frequency coupling to behavioural enhancements and decrements as a function of adaptive motor control. While the existing literature has provided substantial insight on internal model updating when major changes in the motor response are required (e.g. motor inhibition), the ability to adapt to more ecologically valid shifts in environmental parameters to maintain the same movement kinematics was poorly understood. Ultimately, our data provides novel mechanistic insight indicating that the adaptive control of movement is governed by strong theta–gamma cross-frequency coupling in the motor cortex. Essentially, during the updating of *identical* movement parameters, stronger coupling during reactive adaptation is necessary for improvements in behaviour, while stronger coupling during expected (i.e. proactive) trials is less essential and led to decrements in performance. Further, while the current study provided insight regarding the oscillatory dynamics serving specific behavioural modifications for reactionary motor control (i.e. coupling-related changes to response distances) future studies will benefit from also evaluating the impact of movement force in the context of proactive–reactive movements to more comprehensively characterize behaviour. Finally, in regard to mechanism, the observed theta–gamma power-to-power coupling observed in our study could be attributable, at least in part, to changes in GABAergic interneuronal pools. Importantly, previous work substantially implicates this GABA-mediated inhibitory drive in modulating local pyramidal cells, giving rise to a host of spectral profiles including the generation and modification of theta and gamma oscillatory activity.^24,68-75^ Thus, while GABA activity was not directly measured in the current study, future work in this area will be critical to fully unravel the nature of oscillatory coupling in the sensorimotor cortices during adaptive motor control.

## Supplementary Material

fcac249_Supplementary_Data

## Abbreviations

BESA =: brain electrical source analysis
DICS =: dynamic imaging of coherent sources
MEG =: magnetoencephalography
M1 =: primary motor

## References

[1] Wolpert DM , Miall RC. Forward models for physiological motor control. Neural Netw. 1996;9(8):1265–1279. 12662535 10.1016/s0893-6080(96)00035-4

[2] Wolpert DM , Ghahramani Z, Jordan MI. An internal model for sensorimotor integration. Science. 1995;269(5232):1880–1882. 7569931 10.1126/science.7569931

[3] Bizzi E , Mussa-Ivaldi FA. Neural basis of motor control and its cognitive implications. Trends Cogn Sci. 1998;2(3):97–102. 21227085 10.1016/s1364-6613(98)01146-2

[4] Rothwell J . Motor Control. 2nd edn. Elsevier; 2004.

[5] Aron AR . From reactive to proactive and selective control: Developing a richer model for stopping inappropriate responses. Biol Psychiatry. 2011;69(12):e55–e68. 20932513 10.1016/j.biopsych.2010.07.024 PMC3039712

[6] Meyer HC , Bucci DJ. Neural and behavioral mechanisms of proactive and reactive inhibition. Learn Mem. 2016;23(10):504–514. 27634142 10.1101/lm.040501.115 PMC5026209

[7] Aron AR , Robbins TW, Poldrack RA. Inhibition and the right inferior frontal cortex. Trends Cogn Sci. 2004;8(4):170–177. 15050513 10.1016/j.tics.2004.02.010

[8] Eagle DM , Baunez C, Hutcheson DM, Lehmann O, Shah AP, Robbins TW. Stop-signal reaction-time task performance: Role of prefrontal cortex and subthalamic nucleus. Cereb Cortex. 2008;18(1):178–188. 17517682 10.1093/cercor/bhm044

[9] Chen X , Scangos KW, Stuphorn V. Supplementary motor area exerts proactive and reactive control of arm movements. J Neurosci. 2010;30(44):14657–14675. 21048123 10.1523/JNEUROSCI.2669-10.2010 PMC2990193

[10] Aron AR , Robbins TW, Poldrack RA. Inhibition and the right inferior frontal cortex: One decade on. Trends Cogn Sci. 2014;18(4):177–185. 24440116 10.1016/j.tics.2013.12.003

[11] Di Russo F , Lucci G, Sulpizio V, et al Spatiotemporal brain mapping during preparation, perception, and action. Neuroimage. 2016;126:1–14. 26608247 10.1016/j.neuroimage.2015.11.036

[12] Zhang F , Iwaki S. Common neural network for different functions: An investigation of proactive and reactive inhibition. Front Behav Neurosci. 2019;13:124. 31231199 10.3389/fnbeh.2019.00124 PMC6568210

[13] Tzagarakis C , Ince NF, Leuthold AC, Pellizzer G. Beta-band activity during motor planning reflects response uncertainty. J Neurosci. 2010;30(34):11270–11277. 20739547 10.1523/JNEUROSCI.6026-09.2010 PMC6633326

[14] Grent-’t-Jong T , Oostenveld R, Jensen O, Medendorp WP, Praamstra P. Competitive interactions in sensorimotor cortex: Oscillations express separation between alternative movement targets. J Neurophysiol. 2014;112(2):224–232. 24760786 10.1152/jn.00127.2014 PMC4064405

[15] Wilson TW , Heinrichs-Graham E, Becker KM. Circadian modulation of motor-related beta oscillatory responses. Neuroimage. 2014;102(Pt 2):531–539. 25128712 10.1016/j.neuroimage.2014.08.013 PMC4252760

[16] Heinrichs-Graham E , Arpin DJ, Wilson TW. Cue-related temporal factors modulate movement-related beta oscillatory activity in the human motor circuit. J Cogn Neurosci. 2016;28(7):1039–1051. 26967947 10.1162/jocn_a_00948 PMC4887290

[17] Pfurtscheller G , Lopes da Silva FH. Event-related EEG/MEG synchronization and desynchronization: Basic principles. Clin Neurophysiol. 1999;110(11):1842–1857. 10576479 10.1016/s1388-2457(99)00141-8

[18] Cassim F , Monaca C, Szurhaj W, et al Does post-movement beta synchronization reflect an idling motor cortex? Neuroreport. 2001;12(17):3859–3863. 11726809 10.1097/00001756-200112040-00051

[19] Jurkiewicz MT , Gaetz WC, Bostan AC, Cheyne D. Post-movement beta rebound is generated in motor cortex: Evidence from neuromagnetic recordings. Neuroimage. 2006;32(3):1281–1289. 16863693 10.1016/j.neuroimage.2006.06.005

[20] Gaetz W , Macdonald M, Cheyne D, Snead OC. Neuromagnetic imaging of movement-related cortical oscillations in children and adults: Age predicts post-movement beta rebound. Neuroimage. 2010;51(2):792–807. 20116434 10.1016/j.neuroimage.2010.01.077

[21] Heinrichs-Graham E , Kurz MJ, Gehringer JE, Wilson TW. The functional role of post-movement beta oscillations in motor termination. Brain Struct Funct. 2017;222(7):3075–3086. 28337597 10.1007/s00429-017-1387-1 PMC5610915

[22] Igarashi J , Isomura Y, Arai K, Harukuni R, Fukai T. A θ-γ oscillation code for neuronal coordination during motor behavior. J Neurosci. 2013;33(47):18515–18530. 24259574 10.1523/JNEUROSCI.2126-13.2013 PMC6618805

[23] Tomassini A , Ambrogioni L, Medendorp WP, Maris E. Theta oscillations locked to intended actions rhythmically modulate perception. Elife. 2017;6:e25618. 28686161 10.7554/eLife.25618 PMC5553936

[24] Gaetz W , Edgar JC, Wang DJ, Roberts TPL. Relating MEG measured motor cortical oscillations to resting γ-aminobutyric acid (GABA) concentration. Neuroimage. 2011;55(2):616–621. 21215806 10.1016/j.neuroimage.2010.12.077 PMC3411117

[25] Muthukumaraswamy SD . Functional properties of human primary motor cortex gamma oscillations. J Neurophysiol. 2010;104(5):2873–2885. 20884762 10.1152/jn.00607.2010

[26] Wilson TW , Slason E, Asherin R, et al An extended motor network generates beta and gamma oscillatory perturbations during development. Brain Cogn. 2010;73(2):75–84. 20418003 10.1016/j.bandc.2010.03.001 PMC2880229

[27] Wilson TW , Slason E, Asherin R, et al Abnormal gamma and beta MEG activity during finger movements in early-onset psychosis. Dev Neuropsychol. 2011;36(5):596–613. 21667363 10.1080/87565641.2011.555573 PMC3151150

[28] Kaiser J , Birbaumer N, Lutzenberger W. Event-related beta desynchronization indicates timing of response selection in a delayed-response paradigm in humans. Neurosci Lett. 2001;312(3):149–152. 11602332 10.1016/s0304-3940(01)02217-0

[29] Praamstra P , Kourtis D, Nazarpour K. Simultaneous preparation of multiple potential movements: Opposing effects of spatial proximity mediated by premotor and parietal cortex. J Neurophysiol. 2009;102(4):2084–2095. 19657085 10.1152/jn.00413.2009 PMC6007848

[30] Heinrichs-Graham E , Wilson TW. Coding complexity in the human motor circuit. Hum Brain Mapp. 2015;36(12):5155–5167. 26406479 10.1002/hbm.23000 PMC4715608

[31] Fry A , Mullinger KJ, O’Neill GC, et al Modulation of post-movement beta rebound by contraction force and rate of force development. Hum Brain Mapp. 2016;37(7):2493–2511. 27061243 10.1002/hbm.23189 PMC4982082

[32] Gaetz W , Liu C, Zhu H, Bloy L, Roberts TPL. Evidence for a motor gamma-band network governing response interference. Neuroimage. 2013;74:245–253. 23454050 10.1016/j.neuroimage.2013.02.013 PMC3817211

[33] Grent-’t-Jong T , Oostenveld R, Jensen O, Medendorp WP, Praamstra P. Oscillatory dynamics of response competition in human sensorimotor cortex. Neuroimage. 2013;83:27–34. 23796548 10.1016/j.neuroimage.2013.06.051

[34] Heinrichs-Graham E , Hoburg JM, Wilson TW. The peak frequency of motor-related gamma oscillations is modulated by response competition. Neuroimage. 2018;165:27–34. 28966082 10.1016/j.neuroimage.2017.09.059 PMC5826720

[35] Spooner RK , Wiesman AI, Proskovec AL, Heinrichs-Graham E, Wilson TW. Prefrontal theta modulates sensorimotor gamma networks during the reorienting of attention. Hum Brain Mapp. 2020;41(2):520–529. 31621977 10.1002/hbm.24819 PMC7268018

[36] Wiesman AI , Koshy SM, Heinrichs-Graham E, Wilson TW. Beta and gamma oscillations index cognitive interference effects across a distributed motor network. Neuroimage. 2020;213:116747. 32179103 10.1016/j.neuroimage.2020.116747 PMC7231968

[37] Spooner RK , Eastman JA, Wiesman AI, Wilson TW. Methodological considerations for a better somatosensory gating paradigm: The impact of the inter-stimulus interval. Neuroimage. 2020;220:117048. 32544524 10.1016/j.neuroimage.2020.117048 PMC7593607

[38] Spooner RK , Wiesman AI, Wilson TW. Peripheral somatosensory entrainment modulates the cross-frequency coupling of movement-related theta-gamma oscillations. Brain Connect. 2022;12(6):524–537. 34269624 10.1089/brain.2021.0003 PMC9419931

[39] Spooner RK , Arif Y, Taylor BK, Wilson TW. Movement-related gamma synchrony differentially predicts behavior in the presence of visual interference across the lifespan. Cerebral Cortex. 2021;31(11):5056–5066. 34115110 10.1093/cercor/bhab141 PMC8491684

[40] Spooner RK , Taylor BK, Ahmad IM, et al Neural oscillatory activity serving sensorimotor control is predicted by superoxide-sensitive mitochondrial redox environments. Proc Natl Acad Sci U S A. 2021;118(43):e2104569118. 10.1073/pnas.2104569118 PMC8639326 34686594

[41] Arif Y , Embury CM, Spooner RK, et al High-definition transcranial direct current stimulation of the occipital cortices induces polarity dependent effects within the brain regions serving attentional reorientation. Hum Brain Mapp. 2022;43(6):1930–1940. 34997673 10.1002/hbm.25764 PMC8933319

[42] Arif Y , Spooner RK, Heinrichs-Graham E, Wilson TW. High-definition transcranial direct current stimulation modulates performance and alpha/beta parieto-frontal connectivity serving fluid intelligence. J Physiol. 2021;599(24):5451–5463. 34783045 10.1113/JP282387 PMC9250752

[43] Spooner RK , Taylor BK, L’Heureux E, et al Stress-induced aberrations in sensory processing predict worse cognitive outcomes in healthy aging adults. Aging. 2021;13(16):19996–20015. 34410999 10.18632/aging.203433 PMC8436901

[44] Aron AR , Poldrack RA. Cortical and subcortical contributions to stop signal response inhibition: Role of the subthalamic nucleus. J Neurosci. 2006;26(9):2424–2433. 16510720 10.1523/JNEUROSCI.4682-05.2006 PMC6793670

[45] Taulu S , Simola J, Kajola M. Applications of the signal space separation method. IEEE Trans Signal Process. 2005;53(9):3359–3372.

[46] Taulu S , Simola J. Spatiotemporal signal space separation method for rejecting nearby interference in MEG measurements. Phys Med Biol. 2006;51(7):1759–1768. 16552102 10.1088/0031-9155/51/7/008

[47] Uusitalo MA , Ilmoniemi RJ. Signal-space projection method for separating MEG or EEG into components. Med Biol Eng Comput. 1997;35(2):135–140. 9136207 10.1007/BF02534144

[48] Wiesman AI , Wilson TW. Attention modulates the gating of primary somatosensory oscillations. Neuroimage. 2020;211:116610. 32044438 10.1016/j.neuroimage.2020.116610 PMC7111587

[49] Kovach CK , Gander PE. The demodulated band transform. J Neurosci Methods. 2016;261:135–154. 26711370 10.1016/j.jneumeth.2015.12.004 PMC5084918

[50] Ernst M . Permutation methods: A basis for exact inference. Stat Sci. 2004;19(4):10.

[51] Maris E , Oostenveld R. Nonparametric statistical testing of EEG- and MEG-data. J Neurosci Methods. 2007;164(1):177–190. 17517438 10.1016/j.jneumeth.2007.03.024

[52] Gross J , Kujala J, Hamalainen M, Timmermann L, Schnitzler A, Salmelin R. Dynamic imaging of coherent sources: Studying neural interactions in the human brain. Proc Natl Acad Sci U S A. 2001;98(2):694–699. 11209067 10.1073/pnas.98.2.694 PMC14650

[53] Hillebrand A , Singh KD, Holliday IE, Furlong PL, Barnes GR. A new approach to neuroimaging with magnetoencephalography. Hum Brain Mapp. 2005;25(2):199–211. 15846771 10.1002/hbm.20102 PMC6871673

[54] Jensen O , Colgin LL. Cross-frequency coupling between neuronal oscillations. Trends Cogn Sci. 2007;11(7):267–269. 17548233 10.1016/j.tics.2007.05.003

[55] Voytek B , D’Esposito M, Crone N, Knight RT. A method for event-related phase/amplitude coupling. NeuroImage. 2013;64:416–424. 22986076 10.1016/j.neuroimage.2012.09.023 PMC3508071

[56] Tort AB , Komorowski RW, Manns JR, Kopell NJ, Eichenbaum H. Theta-gamma coupling increases during the learning of item-context associations. Proc Natl Acad Sci U S A. 2009;106(49):20942–20947. 19934062 10.1073/pnas.0911331106 PMC2791641

[57] Friese U , Köster M, Hassler U, Martens U, Trujillo-Barreto N, Gruber T. Successful memory encoding is associated with increased cross-frequency coupling between frontal theta and posterior gamma oscillations in human scalp-recorded EEG. Neuroimage. 2013;66:642–647. 23142278 10.1016/j.neuroimage.2012.11.002

[58] Lisman J , Jensen O. The θ-γ neural code. Neuron. 2013;77(6):1002–1016. 23522038 10.1016/j.neuron.2013.03.007 PMC3648857

[59] Roux F , Uhlhaas PJ. Working memory and neural oscillations: α-γ versus θ-γ codes for distinct WM information? Trends Cogn Sci. 2014;18(1):16–25. 24268290 10.1016/j.tics.2013.10.010

[60] von Nicolai C , Engler G, Sharott A, Engel AK, Moll CK, Siegel M. Corticostriatal coordination through coherent phase-amplitude coupling. J Neurosci. 2014;34(17):5938–5948. 24760853 10.1523/JNEUROSCI.5007-13.2014 PMC6608287

[61] Voytek B , Kayser AS, Badre D, et al Oscillatory dynamics coordinating human frontal networks in support of goal maintenance. Nat Neurosci. 2015;18(9):1318–1324. 26214371 10.1038/nn.4071 PMC4551604

[62] Bramson B , Jensen O, Toni I, Roelofs K. Cortical oscillatory mechanisms supporting the control of human social-emotional actions. J Neurosci. 2018;38(25):5739–5749. 29793973 10.1523/JNEUROSCI.3382-17.2018 PMC6595981

[63] Emmerling F , Duecker F, de Graaf TA, Schuhmann T, Adam JJ, Sack AT. Foresight beats hindsight: The neural correlates underlying motor preparation in the pro-/anti-cue paradigm. Brain Behav. 2017;7(5):e00663. 28523216 10.1002/brb3.663 PMC5434179

[64] Busch NA , Dubois J, VanRullen R. The phase of ongoing EEG oscillations predicts visual perception. J Neurosci. 2009;29(24):7869–7876. 19535598 10.1523/JNEUROSCI.0113-09.2009 PMC6665641

[65] Landau AN , Fries P. Attention samples stimuli rhythmically. Curr Biol. 2012;22(11):1000–1004. 22633805 10.1016/j.cub.2012.03.054

[66] Landau AN , Schreyer HM, van Pelt S, Fries P. Distributed attention is implemented through theta-rhythmic gamma modulation. Curr Biol. 2015;25(17):2332–2337. 26279231 10.1016/j.cub.2015.07.048

[67] Cheyne D , Bells S, Ferrari P, Gaetz W, Bostan AC. Self-paced movements induce high-frequency gamma oscillations in primary motor cortex. Neuroimage. 2008;42(1):332–342. 18511304 10.1016/j.neuroimage.2008.04.178

[68] Johnson NW , Özkan M, Burgess AP, et al Phase-amplitude coupled persistent theta and gamma oscillations in rat primary motor cortex in vitro. Neuropharmacology. 2017;119:141–156. 28400257 10.1016/j.neuropharm.2017.04.009

[69] Singer W . Neuronal synchrony: A versatile code for the definition of relations? Neuron. 1999;24(1):49–65, 111–125. 10677026 10.1016/s0896-6273(00)80821-1

[70] Bartos M , Vida I, Jonas P. Synaptic mechanisms of synchronized gamma oscillations in inhibitory interneuron networks. Nat Rev Neurosci. 2007;8(1):45–56. 17180162 10.1038/nrn2044

[71] Fries P , Nikolić D, Singer W. The gamma cycle. Trends Neurosci. 2007;30(7):309–316. 17555828 10.1016/j.tins.2007.05.005

[72] Uhlhaas PJ , Pipa G, Lima B, et al Neural synchrony in cortical networks: History, concept and current status. Front Integr Neurosci. 2009;3:17. 19668703 10.3389/neuro.07.017.2009 PMC2723047

[73] Buzsáki G , Wang XJ. Mechanisms of gamma oscillations. Annu Rev Neurosci. 2012;35(1):203–225. 22443509 10.1146/annurev-neuro-062111-150444 PMC4049541

[74] Uhlhaas PJ , Singer W. Neuronal dynamics and neuropsychiatric disorders: Toward a translational paradigm for dysfunctional large-scale networks. Neuron. 2012;75(6):963–980. 22998866 10.1016/j.neuron.2012.09.004

[75] Vinck M , Womelsdorf T, Buffalo EA, Desimone R, Fries P. Attentional modulation of cell-class-specific gamma-band synchronization in awake monkey area v4. Neuron. 2013;80(4):1077–1089. 24267656 10.1016/j.neuron.2013.08.019 PMC3840396

