# Supplementary material for: Cortical theta–gamma coupling governs the adaptive control of motor commands
Source: Brain Commun. 2022 Oct 6;4(6):fcac249. doi: 10.1093/braincomms/fcac249 (PMC9631971; doi:10.1093/braincomms/fcac249)
Supplement: fcac249_Supplementary_Data [file fcac249_supplementary_data.zip › Supplementary_Video_1_legend.docx]

**Supplementary Material**

**Supplementary Video 1. Video of Proactive Reactive Motor Control Task.** Participants fixated on a centrally-located crosshair as a red dot moved clockwise toward the blue target interval. Participants were instructed to respond with their right index finger when the red dot was in the blue interval (proactive condition: Pro). In the reactive condition (Re), participants were presented with the same proactive stimulus features (i.e., target interval parameters) and performed the same movement, but the blue target interval shrunk and shifted to one of four locations when the red dot reached a fixed distance away from the original target interval (~150 ms). Trial types were presented in pseudorandomized order and the instructions were identical regardless of the target’s location. The video presents the following order of task conditions: Pro, Re, Re, Pro, Re, Pro, Re, Pro.
